# Hormone replacement therapy is associated with gastro-oesophageal reflux disease: a retrospective cohort study

**DOI:** 10.1186/1471-230X-12-56

**Published:** 2012-05-29

**Authors:** Helen Close, James M Mason, Douglas Wilson, A Pali S Hungin

**Affiliations:** 1 Durham Clinical Trials Unit, Queen’s Campus, Wolfson Research Institute, University of Durham, University Boulevard, Stockton-on-Tees, TS17 6BH, UK; 2 School of Medicine and Health, Queen’s Campus, Wolfson Research Institute, University of Durham, University Boulevard, Stockton-on-Tees, TS17 6BH, UK

**Keywords:** Gastro-oesophageal reflux disease, Hormone replacement therapy, Menopause

## Abstract

**Background:**

Oestrogen and progestogen have the potential to influence gastro-intestinal motility; both are key components of hormone replacement therapy (HRT). Results of observational studies in women taking HRT rely on self-reporting of gastro-oesophageal symptoms and the aetiology of gastro-oesophageal reflux disease (GORD) remains unclear. This study investigated the association between HRT and GORD in menopausal women using validated general practice records.

**Methods:**

51,182 menopausal women were identified using the UK General Practice Research Database between 1995–2004. Of these, 8,831 were matched with and without hormone use. Odds ratios (ORs) were calculated for GORD and proton-pump inhibitor (PPI) use in hormone and non-hormone users, adjusting for age, co-morbidities, and co-pharmacy.

**Results:**

In unadjusted analysis, all forms of hormone use (oestrogen-only, tibolone, combined HRT and progestogen) were statistically significantly associated with GORD. In adjusted models, this association remained statistically significant for oestrogen-only treatment (OR 1.49; 1.18–1.89). Unadjusted analysis showed a statistically significant association between PPI use and oestrogen-only and combined HRT treatment. When adjusted for covariates, oestrogen-only treatment was significant (OR 1.34; 95% CI 1.03–1.74). Findings from the adjusted model demonstrated the greater use of PPI by progestogen users (OR 1.50; 1.01–2.22).

**Conclusions:**

This first large cohort study of the association between GORD and HRT found a statistically significant association between oestrogen-only hormone and GORD and PPI use. This should be further investigated using prospective follow-up to validate the strength of association and describe its clinical significance.

## Background

Gastro-oesophageal reflux disease (GORD) is a common relapsing disorder largely caused by repeated exposure in the lower oesophagus to the retrograde flow of gastric contents [1]. Epidemiological studies have shown that reflux is experienced by 3–20% of the population at least weekly [2-4]. It is one of the most prevalent conditions seen in primary care [5,6] and is costly in terms of pharmacological therapy and investigations [5]. Symptoms of GORD, including heartburn, regurgitation and nausea, are associated with a reduced quality of life [7,8].

The aetiology of GORD remains unclear, although research has identified the main risk factors as heredity [9], increased body mass index (BMI) [10] and tobacco smoking [11]. Some studies indicate a stronger association between GORD and obesity in females than males, suggesting a link with female sex hormones [12]. This hypothesis is supported by in-vitro and clinical data which suggest an indirect action of sex hormones on GI motility [13-16], evidence of reduced upper GI motility during the menstrual cycle [17] and reduced oesophageal sphincter pressure during pregnancy [18]. Recent studies suggest that hormone replacement therapy (HRT) may be associated with GORD in post-menopausal women [19]. A recent Swedish cohort study of female twins suggests that oestrogen-only HRT is an independent risk factor for GORD symptoms [20] (OR 1.32; 95% CI 1.18–1.47). A large US randomised controlled trial reported a similar association among post-menopausal women with hysterectomy [21], findings mirrored in a US cohort study of post-menopausal registered nurses [22]. Although a Norwegian case–control study indicated that obesity increases the influence of oestrogen use upon GORD (OR 2.3; 95%CI 1.1–4.8), it is difficult to draw firm conclusions given the small numbers [12]. These findings contrast with in-vitro evidence suggesting that progestogen use may play a greater role than oestrogen in upper GI motility [23]. Furthermore, these European and US studies relied on self-reporting of symptoms and did not take into account time from onset of menopausal symptoms. In light of these results, the goal of this study was to establish the extent of the association between upper GI symptoms and the use of forms of HRT, and the relative importance of oestrogen compared with progestogen in the UK population.

## Methods

A retrospective cohort study was designed and conducted, accessing records from the General Practice Research Database (GPRD). The GPRD provides anonymised access to electronic primary care medical records and prescription data for a representative 6% of the UK population [24], and has been extensively validated as a reliable source of GORD information [25]. Diagnoses were identified using Read codes and prescriptions using Prescription Pricing Authority (PPA) codes; all codes for GORD symptoms, outcomes, medications and covariates are available on request. Data were accessed under the Medical Research Council licence for academic groups (protocol reference 07109).

### Disease definitions

Menopausal symptoms were defined using a wide range of diagnostic and referral codes indicating menopause or menopausal symptoms such as “menopause” and “syndrome menopausal”. Gastro-oesophageal outcomes were categorised in two ways firstly using diagnostic codes specific to GORD (ICD 10 category K21), for example “gastro-oesophageal reflux”, and secondly including Barrett’s Oesophagus and more general codes indicative of symptoms of GORD such as ‘reflux oesophagitis’ and ‘waterbrash’. In recognition of the reported lack of standardisation of GORD recording [25] in general practice; we also investigated the association between proton-pump inhibitor (PPI) and hormone use.

### Cohort selection

The original GPRD cohort contained complete records for 102,602 women with a recorded diagnosis of menopause aged between 40–70 at menopause diagnosis (T_0_). Diagnosis occurred within the cohort window of 01/01/1995 – 31/12/2004 (largely avoiding the subsequent reduction in HRT following reports of increased cancer and cardiovascular risk). Patients with less than 24 months of continuous GP registration were excluded, as were patients with malignancy or pregnancy recorded within the study window. The study window for identifying the cohort was defined as two years from menopause diagnosis (T_0_ +/− 24 months). Patients who commenced HRT more than 2 years before or after the first record of menopause were excluded, thus a cohort of N = 51,182 women with a record of menopause were within the study window. A sub-cohort of hormone users were matched, according to calendar year, age at menopause, socio-economic status of GP practice, and date closest to menopause, with one user for every two non-users.

In addition to GORD, records were evaluated for hysterectomy, osteoarthritis, non-steroidal anti-inflammatory drugs (NSAIDs), bisphosphonates and calcium supplement use at menopause diagnosis. Limited by variable reporting within the GPRD, smoking and alcohol status were crudely categorized by “non-user”, “user” or “ex-user” at menopause. Mean BMI was calculated using all BMI readings (kg/m^2^) within the study window and categorized as underweight (<18.5), normal (18.5 to 24.9), overweight (25 to 29.9), obese (30 to 39.9), morbidly obese (≥40). GP practices were allocated a quintile score for socioeconomic status based in the Index of Multiple Deprivation (IMD) [26].

### Drug exposure definitions

Hormone use incorporated the following proprietary and generic classifications: Combined hormone (conjugated oestrogen with progestogen; estradiol with progestogen); oestrogen-only (oestradiol only; oestradiol, oestriol and oestrone; oestriol only; oestropipate only; conjugated oestrogens only); tibolone; and progestogen (other than for contraception; dydrogesterone; medroxyprogesterone; norethisterone; progesterone). For analytic purposes, the term ‘All hormone (AH)’ incorporates all of the above categories; sensitivity analysis reports on the differences between categories.

Bisphosphonates incorporated bisphosphonates and other drugs affecting bone metabolism including alendronic acid. Calcium supplements comprised calcium gluconate, calcium lactate, and calcium carbonate preparations. Non-steroidal anti-inflammatory drugs comprised all NSAIDs. Exposure to these drugs was defined as at least one prescription record in the time window of 2 years prior to or at menopause diagnosis. Proton pump inhibitors (PPIs) comprised esomeprazole, lansoprazole, omeprazole, pantoprazole, and rabeprazole sodium. Use of PPI (categorised as yes/no) was identified both before and after hormone exposure.

### Analysis

The relative risk of GORD or PPI use was estimated as an odds ratio (OR) for hormone use compared to non use. Models were subsequently adjusted for demographic, comorbidity and drug exposures variables. Data management and manipulation was performed using Stata/IC 10. Binomial logistic regression analyses used SPSS v15; the response variable was the presence or absence of GORD or PPI use, and independent variables were either categorical, ordinal or continuous as appropriate. The analysis fitted odds ratios for each of the independent variables with 95% confidence intervals (CIs). The Wald statistic was used to indicate the statistical significance of each fitted logit coefficient (different from zero) corresponding to each independent variable.

Simple regression examined the unadjusted strength of association between each different form of HRT and GORD. Cases and controls were then matched according to calendar year, age at menopause, socio-economic status of GP practice, and date closest to menopause, and simple matched analysis was conducted. The matched dataset was then used to conduct multiple regression analysis taking into account smoking (never/ever/ex), alcohol (never/ever/ex), BMI (subdivided as <18.5, 18.5–24.9, 25–29.9, 30–39.9) and current drug use (NSAID use, calcium supplements and bisphosphonate, duration subdivided into <30 days, and ≥30 days). After extensive exploration, five models for PPI-use and for GORD response were chosen for further detailed analysis on the basis that they allowed for full exploration of the relative effects of risk factors. Model A featured simple regression (unadjusted estimates). Models B to E featured multiple regression (adjusted estimates). Model B incorporated NSAID use (never/ever) calcium use (never/ever), bisphosphonate use (never/ever), BMI, alcohol, and smoking; Model C incorporated NSAID use (never/ever), calcium use (never/ever), bisphosphonates (never/ever); Model D incorporated Model B but exchanged never/ever drug use for drug duration (<30 days, and ≥30 days) plus BMI, alcohol and smoking; Model E incorporated Model D minus BMI, alcohol and smoking. All subgroup analyses were prospectively planned, informed by previous research and clinical expertise. Smoking, alcohol, and BMI had high proportions of missing or unreliable (out of range) data; the final models reported did not include these variables.

## Results

Among 51,182 women with medical records taken from 414 general practices, 22,101 women (43%) were hormone users and 29,081 (57%) were non-users. Overall, users and non-users had clinically similar baseline demographic characteristics, although non-users of hormones were slightly less likely to use NSAIDs, and a higher proportion of oestrogen-only users had undergone hysterectomy (Table 1). A total of 23,210 women (45%) had a record of hysterectomy while 21,835 women (43%) had at least one record of pregnancy ever. The mean age of commencing hormone replacement was 49.7 years and the mean exposure duration was 5.4 years. The most common hormone replacement was combined oestrogen and progestogen; 68.3% of women were exposed to more than one type of hormone and 1% of users stopped taking hormone after one prescription.

**Table 1:** Cohort baseline at menopause diagnosis according to use or non-use of hormone

**Characteristics**		**Oestrogen Users N = 7678)**	**Tibolone Users N = 1539**	**Combined Hormone users N = 9137**	**Progestogen Users N = 3747**	**Non-users N = 29081 (%)**
**Age (y)**	40 to 49	2987 (39%)		275 (18%)	3941 (43%)	2212 (59%)	9795 (34%)
	50 to 59	3229 (42%)		902 (59%)	4464 (49%)	1348 (36%)	13189 (45%)
	60 to 70	1462 (19%)		362 (24%)	732 (8%)	187 (5%)	6097 (21%)
	Mean (SD)	52.6 (7.23)		55.1 (6.16)	50.9 (5.54)	49.1 (5.44)	53.4 (7.24)
**Alcohol user**^**1**^	Non-user	583 (16%)		104 (14%)	617 (14%)	279 (16%)	2243 (17%)
	User	3097 (83%)		615 (85%)	3840 (85%)	1437 (83%)	10386 (81%)
	Ex-user	69 (2%)		7 (1%)	57 (1%)	20 (1%)	197 (2%)
**Smoker**^**2**^	Non-user	2526 (53%)		501 (54%)	2966 (52%)	1208 (54%)	9719 (59%)
	User	1452 (30%)		279 (30%)	1817 (32%)	720 (32%)	4010 (24%)
	Ex-user	800 (17%)		149 (16%)	955 (17%)	317 (14%)	2766 (17%)
**BMI**^**3**^	Underweight	76 (2%)		13 (1%)	86 (2%)	24 (1%)	222 (1%)
	Normal	1928 (41%)		374 (40%)	2524 (44%)	833 (39%)	5771 (37%)
	Overweight	1606 (34%)		338 (36%)	1943 (34%)	627 (30%)	5038 (32%)
	Obese	1004 (21%)		194 (21%)	1034 (18%)	530 (25%)	3812 (25%)
	Morbidly Obese	121 (3%)		25 (3%)	122 (2%)	104 (5%)	702 (5%)
	Mean (SD)	26.9 (5.47)		27.1 (5.80)	26.4 (5.17)	27.7 (6.39)	27.7 (6.11)
**Practice IMD**^**4**^	0	1759 (23%)		311 (20%)	1988 (22%)	832 (22%)	6112 (21%)
	1	1252 (16%)		248 (16%)	1609 (18%)	702 (19%)	5287 (16%)
	2	1578 (21%)		255 (17%)	1804 (20%)	764 (20%)	5743 (20%)
	3	1453 (19%)		344 (22%)	1751 (19%)	731 (20%)	5757 (20%)
	4	1636 (21%)		381 (25%)	1985 (22%)	718 (19%)	6182 (21%)
**Hysterectomy**		3470 (45%)		165 (11%)	462 (5%)	472 (13%)	3229 (11%)
**NSAID use**		4191 (55%)		879 (57%)	4750 (52%)	2242 (60%)	13993 (48%)
**Bisphophonates**		188 (2%)		57 (4%)	192 (2%)	53 (2%)	827 (3%)
**Calcium supplements**		419 (5%)		118 (8%)	477 (5%)	113 (3%)	1707 (6%)
**GORD symptoms**^**5**^		1915 (25%)		387 (25%)	2031 (22%)	859 (23%)	6689 (23%)
**PPI use**^**5**^		564 (7%)		88 (6%)	555 (6%)	239 (6%)	1947 (7%)

^1^54% of patients had missing data.

^2^41% of patients had missing data.

^3^43% of patients had missing data.

^4^Index of Multiple Deprivation (IMD) based on practice post-code. Quintile 0 is the least deprived, quintile 4 is the most deprived.

^5^Prior to menopause.

A total of 42,724 (84%), women had ever been recorded as having GORD symptoms, with a total of 18.5% of all consultations coded as ‘Dyspepsia’, and 3.3% of consultations coded as ‘Gastro-oesophageal Reflux Disease’. The majority of these occurrences were post-menopause. Prior to the study window (pre-menopause), overall 11881 women (23%) consulted their GP for GORD symptoms; GORD reporting rates were comparable between groups prior to hormone exposure (25% of oestrogen-only and tibolone users reported GORD prior to hormone exposure compared to 22% of combined hormone and 23% of progestogen users and non-hormone users).

Table 2 shows the simple analysis (unadjusted for other patient characteristics) of each form of hormone and the strength of their association with GORD, comparing unmatched with matched data. All forms of hormone were statistically significantly associated with reported GORD symptoms (Table 2) both in the unmatched and the matched groups.

**Table 2:** Risk of GORD and PPI use among hormone replacement therapy users: simple regression

		**All hormone users**	**Oestrogen-only users**	**Tibolone users**	**Combined HRT users**	**Progestogen users**
No. of hormone users	*Unmatched cohort*	22101	9137	7678	1539	3747
	*Matched*^*1*^*cohort*	2777	950	167	1190	470
GORD OR (95%CI) p	*Unmatched cohort*	1.23 (1.18–1.27) <0.001	1.36 (1.29–1.43) <0.001	1.26 (1.23–1.51) <0.001	1.15 (1.09–1.20) <0.001	1.11 (1.04–1.20) 0.040
	*Matched cohort*	1.29 (1.12–1.48) <0.001	1.59 (1.27–2.0) <0.001	1.14 (1.06–1.22) <0.001	1.14 (0.92–1.43) 0.234	1.15 (1.06–1.23) <0.001
PPI^2^ OR (95%CI) ^3^p	*Unmatched cohort*	1.38 (1.19–1.60) <0.001	1.46 (1.16–1.84) 0.001	1.52 (0.92–2.50) 0.100	1.41 (1.10–1.80) 0.006	1.06 (0.71–1.59) 0.760
	*Matched cohort*	1.30 (1.15–1.52) 0.001	1.42 (1.10–1.84) 0.007	1.38 (0.48–3.95) 0.549	1.21 (0.95–1.54) 0.121	1.18 (0.72–1.95) 0.512

1. Uses and non-users were matched according to calendar year, age at menopause, socio-economic status of GP practice, and date closest to menopause.

2. Ever PPI use within study window.

3. P-values: comparison of binary variables by adjusted *χ*^2^ test; continuous variables by Student’s *t*-test; multiple category variables by *χ*^2^ test adjusted for trend.

Adjusted analyses (shown in Table 3) explored the relative strength of association between different forms of hormone and GORD, taking into account certain patient characteristics. This showed a statistically significant independent association between oestrogen-only use and GORD (OR 1.49, 95% CI 1.18–1.89, p = 0.001) when taking into account the relative effect of other risk factors (NSAID, bisphosphonate and calcium use). Other unadjusted associations between hormone therapies and GORD did not persist when models were adjusted for these risk factors. Previously known independent risk factors for GORD were confirmed; the models provide evidence of the independent but varying influence of NSAID use and calcium on GORD.

**Table 3:** Risk of GORD among hormone replacement users: multiple regression

	**Oestrogen-only users** No. of GORD cases OR (95% CI) P	**Tibolone users** No. of GORD cases OR (95% CI) P	**Combined HRT users** No. of GORD cases OR (95% CI) P	**Progestogen users** No. of GORD cases OR (95% CI) P
GORD				
Hormone	144	21	137	56
Non-hormone	572	572	572	572
OR (95% CI) p	1.49 (1.18–1.89) 0.001	0.79 (0.45–1.4) 0.419	1.09 (0.87–1.37) 0.445	1.29 (0.90–1.84) 0.170
NSAIDs <30d				
Hormone	23	3	22	12
Non-hormone	99	99	99	99
OR (95% CI) p	1.52 (1.09–2.12) 0.014	1.08 (0.48–2.48) 0.848	1.25 (0.92–1.71) 0.150	1.09 (0.68–1.74) 0.718
NSAIDs ≥30d				
Hormone	75	14	62	21
Non-hormone	233	233	233	233
OR (95% CI) p	2.06 (1.59–2.66) < 0.001	3.42 (1.22–9.64) 0.020	1.97 (1.56–2.50) < 0.000	1.14 (0.77–1.68) 0.520
Calcium <30d				
Hormone	1	0	0	0
Non-hormone	10	10	10	10
OR (95% CI) p	0.56 (0.13–2.41) 0.439	1.58 (0.31–8.02) 0.582	0.49 (0.11–2.06) 0.325	4.14 (1.40–12.27) 0.010
Calcium ≥30d				
Hormone	11	2	11	0
Non-hormone	37	37	37	37
OR (95% CI) p	2.15 (1.26–3.66) 0.005	1.88 (0.53–6.72) 0.330	1.99 (1.18–3.36) 0.010	1.64 (0.523–5.07) 0.393
Bisphosphonate <30d				
Hormone	0	0	1	0
Non-hormone	3	3	3	3
OR (95% CI) p	0.56 (0.06–4.80) 0.592	- (−) 0.999	1.11 (0.29–4.26) 0.886	- (−) 0.999
Bisphosphonate ≥30d				
Hormone	6	0	4	0
Non-hormone	12	12	12	12
OR (95% CI) p	0.93 (0.43–2.04) 0.865	0.84 (0.15–4.75) 0.845	0.69 (0.27–1.79) 0.445	0.75 (0.13–4.27) 0.744

This table shows the number of prospective GORD positive events for hormone replacement use and non-use.

A total of 35639 women (34.7%) were ever-users of PPIs with a mean number of 64 PPI prescriptions. Pre-menopause, the use of PPI prescriptions was comparable across hormone and non-hormone groups for approximately 6% of women.

Simple regression, unmatched and matched analyses (Table 2) showed a consistent statistically significant association between PPI use and oestrogen-only use only (OR 1.46, p = 0.001 and OR 1.42, p = 0.007 respectively). This association remained present in adjusted analysis (OR 1.34, 95% 1.03–1.74, p = 0.027, Table 4). In unadjusted models, the association between GORD or PPI use and progestogen was not statistically significant, however, the association was significant (OR 1.50, 95% CI 1.01–2.22, p = 0.044) in the adjusted analysis. The previously known association between NSAID use and PPI use was confirmed but small numbers of users of calcium supplements and bisphosphonates gave non-significant findings.

**Table 4:** Risk of PPI use among hormone replacement users: multiple regression

	**Oestrogen-only users** No. of GORD cases OR (95% CI) P	**Tibolone users** No. of GORD cases OR (95% CI) P	**Combined HRT users** No. of GORD cases OR (95% CI) P	**Progestogen users** No. of GORD cases OR (95% CI) P
PPI				
Hormone	110	21	116	49
Non-hormone	467	467	467	467
OR (95% CI) p	1.34 (1.03–1.74) 0.027	0.76 (0.43–1.37) 0.367	1.15 (0.90–1.47) 0.250	1.50 (1.01–2.22) 0.044
NSAIDs <30d				
Hormone	22	2	18	10
Non-hormone	81	81	81	81
OR (95% CI) p	1.57 (1.09–2.26) 0.016	0.83 (0.34–2.08) 0.700	1.57 (1.13–2.19) 0.008	1.15 (0.67–1.98) 0.606
NSAIDs ≥30d				
Hormone	56	16	58	24
Non-hormone	200	200	200	200
OR (95% CI) p	2.02 (1.52–2.69) < 0.001	2.55 (1.41–4.63) 0.002	2.37 (1.82–3.07) < 0.001	1.61 (1.05–2.46) 0.029
Calcium <30d				
Hormone	1	1	1	0
Non-hormone	11	11	11	11
OR (95% CI) p	0.97 (0.28–3.35) 0.959	2.78 (0.64–12.05) 0.172	1.21 (0.41–3.55) 0.732	3.60 (1.12–11.56) 0.031
Calcium ≥30d				
Hormone	12	2	10	0
Non-hormone	29	29	29	29
OR (95% CI) p	1.98 (1.12–3.50) 0.019	2.9 (0.90–9.43) 0.075	1.21 (0.65–2.26) 0.555	1.85 (0.59–5.82) 0.293
Bisphosphonate <30d				
Hormone	1	0	0	0
Non-hormone	3	3	3	3
OR (95% CI) p	3.05 (0.70–13.42) 0.139	- (−) 0.999	- (−) 0.999	1.50 (0.14–15.69) 0.737
Bisphosphonate ≥30d				
Hormone	6	2	7	0
Non-hormone	18	18	18	18
OR (95% CI) p	1.96 (0.94–4.12) 0.074	1.70 (0.40–7.19) 0.474	2.01 (0.93–4.73) 0.075	1.38 (0.29–6.60) 0.685

This table shows the number of prospective PPI users for hormone replacement use and non-use.

Preliminary unadjusted models including BMI, alcohol and smoking had large numbers of missing data. These variables poorly fitted any model of GORD or PPI use in unadjusted analyses. When adjusted models of GORD were analysed with BMI, alcohol and smoking as independent variables, similarly none fitted at a statistically significant level. In unadjusted analysis, there was a small but unimportant association between BMI and PPI use (OR 1.04, 95% CI 1.01–1.056, p = 0.001). Alcohol use (OR 4.17, 95% CI 1.20–14.52, p = 0.025) was an additional independent factor in a model of PPI use among combined hormone users, this may be a chance finding in a model involving small numbers.

## Discussion

Within the range of models tested, there was a consistent association between GORD or PPI-use and oestrogen-only hormone replacement. Our evaluation represents the first direct controlled comparison of hormone types for a clinically meaningful GORD diagnosis in a UK population, allowing for multiple hypothesis testing. The large sample size enabled extensive model refinement and subgroup analyses in three ways. Firstly, simple analyses allowed us to identify the potential association between different forms of HRT and GORD regardless of other patient characteristics. This was the first step in understanding the differences between forms of HRT and showed that oestrogen-only HRT presented a possible association with GORD. Secondly, adjusted analysis allowed us to confirm that, even when accounting for known risk factors (NSAID use and other medications), there was an association between oestrogen-only HRT and GORD. Thirdly, we matched cases with controls according to year, age, and socio-economic status in order to account for the effect of these variables upon the incidence of GORD. This further supports the possible association between oestrogen-only HRT and GORD. This study is also the first to compare progestogen only with hormone replacement therapy. Although progestogen was independently associated with PPI use, this was not consistent across simple and adjusted analyses or across GORD groups but the reasons for this are unclear. It was previously thought that progestogen may have a relaxing effect on lower oesophageal sphincter tone. More recent work suggests that oestrogen increases nitric oxide synthesis, which results in smooth muscle relaxation in both human [27] and animal models [28] and may, therefore, be involved in the pathogenesis of GORD. Our findings suggest that oestrogen-only hormone has a stronger independent association with GORD than progestogen, refuting earlier in-vitro studies suggesting progestogen was the most important hormone in GORD aetiology.

There is a well established association between NSAIDs and GORD, which our study suggests may be higher in magnitude with tibolone use although our findings are not statistically significant. Tibolone has oestrogenic, progestogenic, and androgenic effects which act to prevent bone loss. It has been associated with decreased levels of fibrinogen, factor VII, plasminogen activator inhibitor 1, homocysteine, and tissue plasminogen activator and with increased levels of C-reactive protein, antithrombin III, and D-dimer [29]. However, it is unclear which of these, if any, may be a contributory factor in the possible interaction between NSAIDs and tibolone. This study did not assess whether the effects of NSAIDs on tibolone users and their risk of GORD were mediated by the type of NSAID drug. Naproxen is thought to be more gastrotoxic than ibuprofen and diclofenac; whether this holds true for the potential interaction with tibolone remains to be established. In this study, the term GORD relates to a range of GORD like symptoms; it is possible to suggest that the association between NSAID and GORD may be related to gastrotoxicity, and not to an increase in ‘true’ GORD.

The use of HRT declined following publicity about its possible association with cancer and cardiovascular disease [30,31]. However, these data go some way to explain the risk of GORD and PPI use in this group and also provide a possible explanation for those now on HRT who may have GORD symptoms. The GP records used in this study may be a more reliable mechanism for recording GORD symptoms than self-reporting methods used in other studies; this may explain the slightly higher level of risk identified in this study compared to other cohort studies [32]. Our findings are likely to be an underestimate of the extent of the problem as many women who chose not to consult for GORD may simply have stopped taking hormone therapy and would therefore be excluded or miscoded in our analysis. Many women might have also have wanted to avoid polypharmacy. Up to 75% of women choose to stop using HRT in the first six months [33] as a result of reported side effects including weight gain, headache, nausea and perceptions of disease risk. While the design of most studies prevents further analysis of side effects [30], it is possible that at least a proportion of these were attributable to GORD. Our findings, if validated in a prospective study, may have important consequences for the management and resources used by patients with upper GI symptoms as these patients would normally constitute a higher use group for acid suppression therapy.

### Study limitations

The GPRD inconsistently records endoscopy findings; because of the lack of secondary care data to verify the presence of GORD, there is a potential risk of misclassification. The current study explored the relationship between HRT and GORD as presenting to and clinically diagnosed by GPs. This is increasingly the only relevant definition at a time when GPs no longer routinely refer patients without alarm signs for endoscopy because of the perceived balance of risks and benefits to these low-risk patients. Because of well acknowledged difficulties in classifying GORD, this study compared a broad definition of GORD encompassing symptoms such as waterbrash, with a more specific definition of ICD 10 codes (K21) indicative of a GORD diagnosis. Simple regression was conducted using both definitions: the similarity of findings strengthens the conclusions. The GPRD records used in this study may in fact be a more reliable mechanism for recording GORD symptoms than self-reporting methods used in other studies.

Given the weaknesses inherent in GORD recording, we also investigated PPI use in this cohort. Although most of the PPI users had a record of GORD, PPI has many other uses. In addition, many patients with GORD were not prescribed PPIs and it is likely that many women suffering side-effects of HRT would not immediately commence PPIs. We therefore treated GORD sufferers and PPI users as two separate ‘proxy’ groups. The fact that findings were similar across these groups strengthens the association between oestrogen-only HRT and GORD.

When seeking causative associations, weaknesses inherent in all observational design are the problem of unmeasured variables, and the difficulties in teasing out temporal relationships. The similarity of hormone users and non-users at baseline and the consistency of effect with different model formulations offers some protection against this. Selection bias is another common threat to validity although in this study GPRD data was collected from a wide cross-section of general practices from across the UK. It is not possible to rule out some underlying factor selecting patients who do and don’t receive HRT which is GORD-related, although there is no evidence for this.

A further potential weakness of any GPRD study is the incompleteness of BMI, smoking and alcohol data due to the fact that most general practices do not routinely and systematically collect this data. For example, only 41% of women had any BMI record within the study window (erroneous range 0.5 to 896000); after data cleaning, only 21% of BMI records were within acceptable limits. The association between these variables and GORD are well established and so in order to understand their relative effect in combination with HRT use, we conducted sub-group analysis on complete cases. Our findings showed that the strength of association with HRT remained the same as in the wider group but because numbers were small, a prospective study design would be required to confirm these findings.

A further limitation of the study, that might weaken the strength of association, is the lack of a direct measure of adherence to prescribed medication. Since HRT may be used in varying doses over time for symptom control, dose dependency could not be adequately examined using this data. We identified outcomes using diagnostic codes but we were unable to assess the severity of GORD symptoms or to assess whether compliance with HRT was affected by the occurrence of GORD. Similarly it was not possible to explore the association between different hormone replacement therapies and the dose or frequency of PPI use in a meaningful way.

## Conclusions

There is a statistically significant independent association between oestrogen-only hormone and GORD and PPI use. Oestrogen-only hormone has a stronger independent association with GORD than progestogen, refuting earlier in-vitro studies suggesting progestogen was the most important hormone in GORD aetiology. The known association between NSAID and GORD appears higher in magnitude with tibolone use than other hormone use but was not found to be statistically significant. This should be further investigated using prospective follow-up to examine the strength of association and describe its clinical significance.

## Competing interest

APSH and JMM have advised and received grants from manufacturers of reflux management products. APSH is a member of the Independent Advisory Committee of the General Practice Research Database (GPRD).

## Authors’ contributions

HC co-designed the study, led data management, participated in data analysis and interpretation, and drafted this manuscript; JMM co-designed the study, participated in data analysis and interpretation, and drafted this manuscript; DW led data analysis and interpretation, and drafted this manuscript; APSH conceived of the study, participated in its design and coordination and drafted this manuscript. All authors read and approved the final manuscript.

## Funding body

European Society for Primary Care Gastroenterology.

## Pre-publication history

The pre-publication history for this paper can be accessed here:

http://www.biomedcentral.com/1471-230X/12/56/prepub

## References

[B1] Klauser AG Schindlbeck N Muller-Lissner SA Symptoms in gastro-oesophageal reflux disease Lancet 1990 335 8683 205 208 10.1016/0140-6736(90)90287-F 1967675

[B2] Ruth M Mansson I Sandberg N The prevalence of symptoms suggestive of esophageal disorders Scand J Gastroenterol 1991 6 73 81 2006401 10.3109/00365529108996486

[B3] Locke GR Talley NJ Fett SL Prevalence and clinical spectrum of Gastroesophageal reflux: a population based study in Olmsted County, Minnesota Gastroenterology 1997 112 1448 1456 10.1016/S0016-5085(97)70025-8 9136821

[B4] He J Ma X Zhao Y Wang R Yan X Yan H Yin P Kang X Fang J Hao Y Li Q Dent J Sung J Zou D Wallander M Johansson S Liu W Li Z A population-based survey of the epidemiology of symptom-defined gastroesophageal reflux disease: the Systematic Investigation of Gastrointestinal Diseases in China Gastroenterology 2010 10 94 20707933 10.1186/1471-230X-10-94 PMC2933714

[B5] Mason J Hungin APS Review article: GORD – the health economic implications Aliment Pharmacol Ther 2005 22 Suppl. 1 20 31 16042656 10.1111/j.1365-2036.2005.02606.x

[B6] Younes Z Johnson DA Diagnostic evaluation in gastroesophageal reflux disease Gastroenterol Clin N Am 1999 28 809 830 10.1016/S0889-8553(05)70091-1 10695003

[B7] Eloubeidi MA Provenzale D Health-related quality of life and severity of symptoms in patients with Barrett’s esophagus and gastroesophageal reflux disease patients without Barrett’s esophagus Am J Gastroenterol 2000 95 1881 1887 10.1111/j.1572-0241.2000.02235.x 10950030

[B8] Solaymani-Doraran M Logan RF West J Mortality associated with Barrett’s esophagus and gastroesophageal reflux disease diagnoses-a population-based cohort study Am J Gastroenterol 2005 100 2616 2621 10.1111/j.1572-0241.2005.00340.x 16393209

[B9] Cameron AJ Lagergren J Henriksson C Gastrooeophageal reflux disease in monozygotic and dizygotic twins Gastroenterology 2002 122 55 59 10.1053/gast.2002.30301 11781280

[B10] Ruigomez A Garcia Rodriguez LA Wallander MA Natural history of gastro-oesophageal reflux disease diagnosed in general practice Aliment Pharmacol Ther 2004 20 751 760 10.1111/j.1365-2036.2004.02169.x 15379835

[B11] Nilsson M Johnsen R Ye W Lifestyle related risk factors in the aetiology of gastro-oesophageal reflux Gut 2004 53 1730 1735 10.1136/gut.2004.043265 15542505 PMC1774312

[B12] Nilsson M Johnsen R Ye W Obesity and estrogen as risk factors for gastroesophageal reflux symptoms Jama 2003 290 66 72 10.1001/jama.290.1.66 12837713

[B13] Smout AJPM Champion MC, Orr WC Gastro-oesophageal reflux disease: Pathogenesis and Diagnosis Evolving concepts in Gastrointestinal Motility 1996 Blackwell Science, Oxford, England 46 63

[B14] Van Thiel DH Gavaler JS Stremple J Lower esophageal sphincter pressure in women using sequential oral contraceptives Gastroenterology 1976 71 232 234 939383

[B15] Singh S Poulsom R Longcroft JM Is cyclical bowel habit mediated by female sex hormones? Eur J Gastroenterol Hepatol 1994 6 925 930 10.1097/00042737-199410000-00015

[B16] Bruce L Faiz B Progesterone effects on three regional gastrointestinal tissues Life Sci 1979 25 729 734 10.1016/0024-3205(79)90515-0 491852

[B17] Turnbull GK Thompson DG Day S Relationship between symptoms, menstrual cycle and orocaecal transit in normal and constipated women Gut 1989 30 30 34 10.1136/gut.30.1.30 2920923 PMC1378226

[B18] Van Thiel DH Wald A Evidence refuting a role for increased abdominal pressure in the pathogenesis of the heartburn associated with pregnancy Am J Obstet Gynaecol 1981 140 420 422.3 10.1016/0002-9378(81)90037-5 7246657

[B19] Menon S Trudgill N Is hormone replacement therapy associated with reflux oesophagitis, Barrett’s oesophagus and oesophageal cancer? Gut 2010 59 Suppl III A117

[B20] Nordenstedt H Zheng Z Cameron AJ Postmenopausal hormone therapy as a risk factor for gastroesophageal reflux symptoms among female twins Gastroenterology 2008 134 4 921 928 10.1053/j.gastro.2008.01.009 18294635 PMC2359826

[B21] Zheng Z Margolis KL Liu S Effects of estrogen with and without progestin and obesity on symptomatic gastroesophageal reflux Gastroenterology 2008 135 1 72 81 10.1053/j.gastro.2008.03.039 18502208 PMC2725519

[B22] Jacobson BC Moy B Colditz GA Postmenopausal hormone use and symptoms of gastroesophageal reflux Arch Intern Med 2008 168 16 1798 1804 10.1001/archinte.168.16.1798 18779468 PMC2761884

[B23] Alvarez-Sánchez A Rey E Achem SR Does progesterone fluctuation across the menstrual cycle predispose to gastroesophageal reflux? Am J Gastroenterol 1999 6 1468 1471 10.1111/j.1572-0241.1999.01128.x 10364009

[B24] The General Practice Research Database, www.gprd.com

[B25] El-Serag-Hill C Jones R Systematic review: the epidemiology of gastro-oesophageal reflux disease in primary care, using the UK General Practice Research Database Aliment Pharmacol Ther 2009 29 470 480 10.1111/j.1365-2036.2008.03901.x 19035977

[B26] Communities and Local Government. Communities and neighbourhoods, Indices of Deprivation 2008: PCT Summaries ID 2008,

[B27] Zervou S Klentzeris LD Old RW Nitric oxide synthase expression and steroid regulation in the uterus of women with menorrhagia Mol Hum Reprod 1999 5 1048 1054 10.1093/molehr/5.11.1048 10541567

[B28] Nuedling S Kahlert S Loebbert K 17-Beta-estradiol stimulates expression of endothelial and inducible NO synthase in rat myocardium in-vitro and in-vivo Cardiovasc Res 1999 43 666 674 10.1016/S0008-6363(99)00093-0 10690338

[B29] Cummings SR Etttinger B Delmas PD The effects of tibolone in older postmenopausal women NEJM 2008 359 697 708 10.1056/NEJMoa0800743 18703472 PMC3684062

[B30] Bakken K Eggen AE Lund E Side-effects of hormone replacement therapy and influence on pattern of use among women aged 45–64 years. The Norwegian Women and Cancer (NOWAC) study 1997 Acta Obstetricia et Gynecologica Scandinavica 2004 83 9 850 856 15315597 10.1111/j.0001-6349.2004.00560.x

[B31] Hall P Ploner A Bjohle J Hunag F Lin C Liu E Miller L Nordgren H Pawitan Y Shaw P Skoog L Smeds J Wedren S Ohd J Bergh J Hormone-replacement therapy influences gene expression profiles and is associated with breast-cancer prognosis: a cohort study BMC Medicine 2006 4 16 10.1186/1741-7015-4-16 16813654 PMC1555602

[B32] Talley NJ Weaver Al Zinsmeister AR Onset and disappearance of gastrointestinal symptoms and functional gastrointestinal disorders Am J Epidemiol 1992 136 165 177 1415139 10.1093/oxfordjournals.aje.a116483

[B33] Studd J HRT and long-term compliance: The efficacy and safety of Menorest Int J Gynecol Obstet 1996 52 Suppl 1 S21 S25 8666123

